# Individual Risk Factors of Mycetoma Occurrence in Eastern Sennar Locality, Sennar State, Sudan: A Case-Control Study

**DOI:** 10.3390/tropicalmed7080174

**Published:** 2022-08-10

**Authors:** Rowa Hassan, Kebede Deribe, Hope Simpson, Stephen Bremner, Osama Elhadi, Mustafa Alnour, Ahmed Hassan Fahal, Melanie Newport, Sahar Bakhiet

**Affiliations:** 1 Mycetoma Research Centre, Soba University Hospital, University of Khartoum, Khartoum 11111, Sudan; 2 Department of Global Health and Infection, Brighton and Sussex Medical School, Brighton BN1 9PX, UK; 3 Children’s Investment Fund Foundation, Addis Ababa P.O. Box 9086, Ethiopia; 4 School of Public Health, College of Health Sciences, Addis Ababa University, Addis Ababa P.O. Box 9086, Ethiopia; 5 Department of Disease Control, London School of Hygiene and Tropical Medicine, London WC1E 7HT, UK; 6 Department of Primary Care and Public Health, Brighton and Sussex Medical School, Brighton BN1 9PX, UK; 7 Department of Radiography, Faculty of Medicine, University of Khartoum, Khartoum 11111, Sudan

**Keywords:** case-control study, mycetoma, risk factors, determinants, Sennar, Sudan

## Abstract

Mycetoma is a serious chronic subcutaneous granulomatous inflammatory disease that is endemic in tropical and subtropical regions, where it impacts profoundly on patients, families, and communities. Individual-level risk factors for the disease are poorly understood. To address this, a case-control study was conducted based on data collected from 60 villages in Eastern Sennar Locality, Sennar State, Sudan. Based on the presence of swelling in any part of the body, or sinus formation with or without grain discharge evident from the lesion by ultrasound examination, we diagnosed 359 cases of mycetoma. For each case, we included three healthy sex-matched persons, with no evidence of mycetoma, from the same village as the control group (n = 1077). The odds for mycetoma were almost three times higher in individuals in the age group 16–30 years (Adjusted Odds Ratio (AOR) = 2.804, 95% CI = 1.424–5.523) compared to those in age group ≤ 15 years. Other factors contributing to the odds of mycetoma were history of local trauma (AOR = 1.892, 95% CI = 1.425–2.513), being unmarried (AOR = 3.179, 95% CI = 2.339–4.20) and owning livestock (AOR = 3.941, 95% CI = 2.874–5.405). In conclusion, certain factors found to be associated with mycetoma in this study could inform a high index of suspicion for mycetoma diagnosis, which would improve early case detection. Other factors found to be associated could inform the development of an interventional program for mycetoma control in Sudan, including education on healthy farming practices and the risks of puncture wounds for individuals residing in endemic areas. However, this work was conducted in one endemic state, while mycetoma cases occur in all states of Sudan. Replicating this study over a wider area would give a fuller picture of the situation, providing the control program with more comprehensive information on the risk factors for the disease.

## 1. Introduction

Mycetoma is a devastating, neglected tropical disease that occurs in tropical and subtropical regions [1]. It is characterized by painless subcutaneous masses with multiple sinuses draining seropurulent discharge and grain, most frequently affecting the feet and hands, though other parts of the body may be involved [2,3]. The disease progresses to involve the skin, deep tissues, and bone, sometimes resulting in massive tissue destruction leading to significant deformities and disability [4]. The disease is associated with massive morbidity, stigma and reduced economic productivity [4,5]. Mycetoma is reported worldwide and Sudan is considered the most endemic country and, yet, there is no community-based study to feed national control programs with comprehensive information. Mycetoma is commonly reported from Mexico, Venezuela, Mauritania, Senegal, Chad, Ethiopia, Somalia, Yemen, and India [6]. Most of the mycetoma cases in Sudan are attributed to fungal organisms, and *Madurella mycetomatis* is the most common causative agent [7]. While actinomycetoma cases in Sudan are commonly caused by *Streptomyces somaliensis*, *Actinomadura madurae* and *Actinomadura pelletierii* [8].

The geographical distribution of mycetoma depends on a range of environmental factors, such as rainfall, humidity, and temperature [9]. A study conducted in Sudan indicated mycetoma is more likely to occur in arid areas proximal to water sources, soil with low calcium and sodium concentrations, and a variety of thorny tree species [10].

There is controversy on the route of infection in mycetoma; however, subcutaneous traumatic inoculation of the causative organism is the most favored hypothesis, based on current evidence [4].

The disease is most frequently reported in young adults aged 20–40 years, but people of all ages are at risk [9]. Males are reported to be at higher risk, but a community-based study in White Nile, Sudan, showed almost equal risk for both sexes [10,11]. Mycetoma commonly affects those who work in close contact with the environment, such as farmers and shepherds [12]. Affected communities are generally of low socioeconomic status and underdeveloped infrastructure to provide clean water and sanitation [13].

There are currently no dedicated control programs for mycetoma, and the only available tools to reduce the disease burden are early case finding and appropriate management. These are hampered by challenges in the diagnosis and treatment of the disease and knowledge gaps surrounding its incidence, prevalence, and determinants of susceptibility [11,12,13]. Diagnosis requires numerous invasive and time-consuming investigations led by an experienced physician, and available treatment options are limited and expensive, require a long time to effect cure, and have a high recurrence rate [14,15,16,17]. The promotion of early case detection is costly and relies on appropriate public health messaging and community education. Existing knowledge gaps surrounding the route of infection, factors favoring transmission, and determinants of susceptibility to the disease impede the design and implementation of effective prevention strategies [4,18,19,20].

We sought to address this issue using a case-control study to determine risk factors for mycetoma infection in a highly endemic area of Sudan.

## 2. Methods

This population-based case-control study was conducted in Eastern Sennar Locality, Sennar State, central-eastern Sudan, and included 41,176 individuals as total population covered in the study. Eastern Sennar Locality is highly endemic according to surveillance databases from the Mycetoma Research Centre (MRC), a World Health Organization (WHO) Collaborating Centre on Mycetoma, and the reference center for mycetoma management and research in Sudan.

The rationale for the sample size was based on the assumption that the prevalence of mycetoma is five cases per 10,000 population, with the average population size of 935 people in the village, and the population of Eastern Sennar locality of 353,196 people. The calculations suggested that sampling 60 villages would give 80% power of detecting mycetoma at 5% significance level. Cases were identified through an exhaustive survey of 60/292 randomly selected villages in the five administrative units of the locality, described in more detail elsewhere [21]. The survey was conducted from June 2019–July 2019, and every household in these sixty villages was visited. Cases were identified through clinical examination of all individuals by a medical doctor. When an individual was not at home for the examination, the household was revisited. All individuals with swelling of any part of the body or sinus formation, with or without grain discharge, were considered suspected cases of mycetoma. All suspected cases were referred to Wad Onsa Regional Mycetoma Centre, where an experienced radiologist performed lesion ultrasound examination to ascertain mycetoma diagnosis. A confirmed mycetoma case was defined as an individual with swelling or sinuses in any body part, with a pocket of fluid containing echogenic grains on ultrasound examination [22].

For each patient, three healthy controls were selected by simple random sampling. We considered that a 3:1 ratio of controls to cases would provide sufficient power to the statistical analyses. Controls were matched on community and gender (i.e., 3 controls of the same gender and from the same village were selected for each case identified), and selected from households where no suspected case was detected.

After village leaders and the study population were informed about the survey objectives and process, all households in the study villages were visited. The questionnaire was written in English and was validated by a team, including a medical doctor and a statistician at the MRC. Responses were captured through electronic data capture forms through open-source software called Open Data Kit (ODK), which collects, manages, and uses data in resource-constrained environments, running via Android devices. It allows for offline data collection with mobile devices in remote areas.

Household-level data included the type of material of the floor, roof, external walls of the dwelling, and hygiene and sanitation amenities. Individual-level factors included age, sex, marital status, educational level, occupation, swelling, history of trauma and wearing shoes/slippers at home and work. Further details, including clinical features, lesion onset, duration, site, and mycetoma family history, were collected from suspected cases.

Data were sent directly to a server at the MRC and imported to the Statistical Package for Social Sciences, SPSS 25 (SPSS, Chicago, IL, USA) for analysis. Descriptive analysis was performed, and bivariate analysis (chi-square test) was used to identify any statistically significant associations between explanatory variables and the outcome variable (confirmed mycetoma).

Univariate and multivariate analyses were undertaken to assess the strength of association of individual and household-level variables with disease status (i.e., being a mycetoma case or control). A stepwise forward and backward selection procedure was used to select inclusion variables in a conditional logistic regression model. A *p*-value of 0.05 or less was used to enter variables into the model and 0.1 or above for removal from the model. The strength of association of each retained variable with mycetoma was expressed using adjusted odds ratios with their 95% confidence interval (CI). The STROBE case-control reporting guidelines were used in this study [23].

## 3. Results

### 3.1. Demographic Characteristics of Cases and Controls

A total of 1436 individuals (359 mycetoma patients and 1077 controls) were included in the case control analysis. The mean age of confirmed mycetoma patients was 27.0 (SD = 16.3) years, ranging from 1–85 years. The mean age of controls was 37.2 (SD = 16.9) years, ranging from 1–105.

The patient group comprised 174 males (48.5%) and 185 females (51.5%), and the control group 537 (49.9%) males and 540 (51.1%) females. Sixty-seven (18.7%) cases and 236 (21.9%) of controls worked as shepherds or farmers. One hundred and thirty-nine cases (38.7%) and 525 controls (48.7%) were illiterate.

### 3.2. Clinical Characteristics of Cases

In total, 290 cases (80.8%) presented with visible swelling, and 245 (68.2%) had lesions in the lower extremities. Sinuses were present in 138 (38.4%), and, of those, 122 (33.9%) patients had discharge. One hundred and twenty-nine cases (35.9%) had a history of local trauma at the mycetoma site.

Age, history of trauma, marital status, education, raising animals within the household and livestock ownership were strongly associated with the odds of mycetoma (*p*-value < 0.05). Compared to individuals aged 0–15, the odds of mycetoma were more than six times higher in individuals in the age group 16-30 years (OR = 6.467, 95% CI = 3.421–12.224, *p* < 0.001), and around two times higher for those aged 31–45 or 46–60 years. The likelihood of mycetoma in individuals with a history of trauma was 71% higher than in those without (OR = 1.710, 95% CI = 1.323–2.209, *p* < 0.001). The odds of mycetoma in unmarried individuals were over four times that in married individuals (OR = 4.117, 95% CI = 3.151–5.381, *p* < 0.001), while illiterate individuals had lower odds (OR = 0.664, 95% CI = 0.521–0.848, *p* = 0.001). Compared to individuals in other occupations, housewives had increased odds of mycetoma (OR = 4.945, 95% CI = 2.747–8.902, *p* < 0.001), as did those who were desk employees (odds ratio, 1.251, 95% CI = 1.279–3.961, *p* = 0.005). Individuals living in households with animals raised within the dwelling had lower odds (OR = 0.557, 95% CI = 0.435–0.713, *p* < 0.001). Ownership of animals increased the odds of mycetoma by more than two times (OR = 2.15, 95% CI = 1.687–2.742, *p* < 0.001) (Table 1).

### 3.3. Multivariate Analysis

After adjusting for other significant variables, being unmarried had higher odds of mycetoma (AOR = 3.179, 95% CI = 2.339–4.20, *p* < 0.001). The odds of the disease were roughly double for patients with a history of local trauma compared to those without (AOR = 1.892, 95% CI = 1.425–2.513, *p* < 0.001). Those aged 16–30 had higher odds (AOR = 2.804, 95% CI = 1.424–5.523, *p* = 0.003) compared to those aged ≤15 years. Illiterate individuals had lower odds of mycetoma (AOR = 0.685, 95% CI = 0.521–0.900, *p* = 0.007). Individuals who owned animals had higher odds of mycetoma (AOR = 3.914, 95% CI = 2.874–5.405, *p* < 0.001), but those keeping animals within their own dwelling had lower odds of disease (AOR = 0.310, 95% CI = (0.303, 95% CI = 0.220–0.416, *p* < 0.001) (Table 2).

## 4. Discussion

Although mycetoma is a serious disease, inflicting disability and stigma on patients across many parts of the world, there remain essential questions about its epidemiology, particularly the risk factors for the disease. In the current study, patients with confirmed mycetoma were compared to community- and sex-matched controls to identify determinants of risk in the study population. To the best of our knowledge, this is the first population-based case-control study of sociodemographic risk factors for mycetoma, providing evidence likely to apply to other settings and support global control efforts.

One of the risk factors we identified was a history of trauma, which roughly doubled the odds of mycetoma. This finding represents the most substantial epidemiological evidence for the theory that skin trauma may facilitate the inoculation of mycetoma-causative organisms into the subcutaneous tissue; although this does not rule out other possible routes of transmission [24]. This hypothesis is supported by the fact that mycetoma-causative organisms typically reside in the soil and evidence that certain *Acacia* trees, whose thorns may facilitate inoculation, are associated with environmental suitability for the disease [10]. However, previous analyses of mycetoma cases have shown a history of trauma in only a few patients, hypothesized to reflect that the trauma may be minor and pass unnoticed by the patient [11]. Moreover, and due to the nature of work for individuals living in endemic areas, causative organisms can be contracted from the environment through previous injuries. The case-control design we employed, including many patients and community-matched controls, allowed us to demonstrate a significant difference in the history of trauma between these two groups.

Many reports in the literature show that young adults and children are the most affected populations [2,6,25,26]. The data obtained in this study showed that the most common age group affected was the category aged 16–30 years. The higher odds in younger adult groups is likely to reflect the fact that these individuals are more likely to be actively engaged in activities, such as agricultural work and animal grazing, which exposes them to mycetoma-causative agents in endemic areas.

As expected, livestock ownership was a strong risk factor for mycetoma. There are several possible mechanisms by which people who raise animals may be at increased risk of mycetoma. People living in rural areas tend to make animal enclosures from the wood of thorny trees and may be at risk of thorn pricks during the construction and maintenance of these structures [11]. As well as being in close contact with the environment, they are likely to be at higher risk of ticks, which are highly prevalent in domestic animals in the Eastern Sennar Locality and hypothesized to play a role in the transmission of mycetoma causative agents to humans [27]. The evidence for this route of transmission is not conclusive, however. While the DNA of mycetoma-causative organisms has been isolated from ticks, this does not prove they are involved in transmission.

We found that the odds of unmarried individuals contracting mycetoma was four times that of their married counterparts. This is likely to reflect the social stigma of mycetoma, which may mean affected individuals are less likely to get married, or more likely to get divorced if they contract the disease after marriage. In rural communities, early marriage is often considered mandatory and social pressure is put on young adults to get married by their early teen years. If they fail to get married early, this pressure can cause psychological distress in addition to the stress and depression occurring as a result of such a stigmatizing disease [28].

Interestingly, there was no significant difference between literate and illiterate populations and there were increased odds for mycetoma in desk employees, contradicting previous reports in the literature that mycetoma is commonly prevalent among poor communities with low education levels [4]. This may be explained by the fact that in mycetoma-endemic areas, most individuals share the same social and economic activities and behavior irrespective of their educational level due to economic constraints. Another factor may be that schools in rural areas are limited to certain areas, and students often have to walk for long distances, putting them at higher risk of contact with mycetoma-causative organisms. We also observed that housewives and desk employees had increased odds of mycetoma. Regarding housewives, they are usually responsible for the cooking, and, hence, they walk long distances to collect wood to use as fuel. Furthermore, housewives work in farming, especially in the harvesting season, which may mean that they have a similar level of exposure.

There are some limitations to this study. The field-based case definition we used lacks specificity compared to the gold standard techniques of microbiological or PCR confirmation of the causative organisms, which were not feasible within our study setting. Nevertheless, our study is, to our knowledge, the first community-based study to apply clinical examination of all individuals, followed by ultrasound examination to ascertain mycetoma cases. Within the limited timeframe of our study, we were also unable to assess end-line treatment outcomes. Another limitation was that this work was conducted in one endemic state, while mycetoma cases occur in all states of Sudan. The factors we identified may not be applicable to all endemic settings. Finally, some suspect cases were lost to follow-up and were not included in the comparative analysis. However, we have no reason to believe that the absence of these individuals was due to their disease status or other systematic factors, so considered this data to be randomly missing.

## 5. Conclusions

In this population-based case-control study on mycetoma, the first of its kind at this scale, the high number of participants enabled precise estimates for the strength of association of various risk factors with disease.

The results of this study could be applied to inform future control of mycetoma. Efforts to raise awareness among clinicians of mycetoma risk factors, particularly those of age, history of trauma, and ownership of animals, could promote earlier diagnosis and treatment of mycetoma in patients presenting swellings or wounds in endemic regions of Sudan. These factors could also inform the design of health awareness campaigns in communities at risk, educate the population about activities that may put them at risk of the disease and encourage them to present early to health facilities if they experience early signs. In addition, education about hygienic practices after encountering local trauma, including cleaning and disinfecting the injured area to avoid contracting mycetoma, is advocated. Finally, this study adds further evidence for the substantial social impact of the disease and the stigma associated with it, which should not be overlooked in assessments of its global burden.

## Figures and Tables

**Table 1:** Demographic characteristics of confirmed cases and controls, showing unadjusted odds ratios (OR) for mycetoma risk factors (n = 1436).

Characteristic	Cases No. (%)	Controls No. (%)	Crude OR (95% CI)	*p*-Value
**Individual factors**				
**Age group**				
0–15 Years	70 (19.5%)	70 (6.5%)	1.0	
16–30 Years	136 (37.9%)	391 (36.3%)	6.467(3.421–12.224)	<0.001
31–45 years	101 (28.1%)	344 (31.9%)	2.249 (1.262–4.008)	0.006
46–60 Years	37 (10.3%)	175 (16.2%)	1.899(1.055–3.416)	0.032
>60 Years	15 (4.2%)	97 (9.0%)	1.367(0.714–2.617)	0.345
**Sex**				
Female	185 (51.5%)	540 (50.1%)	1.0	
Male	174 (48.5%)	537 (49.9%)	1.069(1.414–2.290)	0.583
**Trauma history**				
Yes	129 (35.9%)	266 (24.7%)	1.710 (1.323–2.209)	<0.001
No	230 (64.1%)	811 (75.3%)	1.0	
**Marital status**				
Married	207 (57.7%)	905 (84.0%)	1.0	
Unmarried	152 (42.3%)	172 (16.0%)	3.864(2.963–5.038)	<0.001
**Education level**				
Literate	220 (61.3%)	552 (51.3%)	1.0	
Illiterate	139(38.7%)	525 (48.7)	0.664 (0.521–0.848)	0.001
**Wear shoes/Slippers**				
Both work and home	261 (72.7%)	828 (76.9%)	1.0	
At work or at home only	32 (9.8%)	84(7.8%)	0.788 (0.574–1.083)	0.142
Not at all	66 (18.4%)	165 (15.3%)	0.952 (0.579–1.566)	0.847
**Occupation**				
Farmers or shepherds	67 (18.7%)	236 (21. 9%)	1.0	
Students	67 (18.7%)	60 (5.6%)	1.257 (0.728–2.170)	0.411
Housewives	114 (31.5%)	447 (41.5%)	4.945 (2.747–8.902)	<0.001
Merchants	15 (4.2%)	76 (7.1%)	1.129 (0.674–1.893)	0.644
Unemployed and underage of work	61 (17.3%)	120 (11.1%)	0.874 (0.422–1.811)	0.717
Desk employee	5 (1.4%)	29 (2.7%)	1.251 (1.279–3.961)	0.005
Freelancer	21 (5.8%)	93 (8.6%)	2.491 (0.969–6.403)	0.058
Other jobs	9 (2.4%)	16 (1.5%)	0.764 (0.264–2.205)	0.618
**Household factors**				
**Source of fuel for cooking**				
Wood and Animal dung	243 (67.7%)	433(40.2%)	0.600 (0.429–0.839)	0.003
Gas and Coal	77 (21.4%)	130 (12.1%)	1.0	
Any source of fuel available	35 (9.7%)	500 (46.4%)	0.403 (0.286–0.569)	<0.001
No food cooked in the house	4 (1.1%)	14 (1.3%)	0.476 (0.151–1.498)	0.205
**Main material of dwelling floor**				
Brick/cement/ceramic	20(5.6%)	50 (4.6%)	2.160 (1.372–3.400)	0.001
Earth/soil and/or sand	326 (90.8%)	1007 (93.5%)	1.0	
Combination	13 (3.6%)	20(1.9%)	1.271 (0.554–2.913)	0.571
**Main material of dwelling roof**				
Traditional/wood/zinc/plastic cover	256(71.3%)	760 (70.6%)	1.101 (0.764–1.588)	0.605
Concrete	42(11.7%)	145(13.5%)	1.0	
Combination	61 (17.0%)	172 (16.0%)	1.169 (0.748–1.826)	0.493
**Main material of dwelling exterior walls**				
Wood	40 (11.1%)	104 (9.7%)	0.824 (0.525–1.294)	0.401
Mud and/or animal dung	114(31.8%)	371 (34.4%)	0.847 (0.626–1.147)	0.284
Red bricks and/or concrete	97 (27.0%)	324 (30.1%)	0.786 (0.572–1.079)	0.137
No walls	108 (30.1%)	278 (25.8%)	1.0	
**Animal raised within the dwelling**				
Yes	127 (35.4%)	534 (49.6%)	0.557 (0.435–0.713)	<0.001
No	232 (64.6%)	543 (50.4%)	1.0	
**Livestock ownership**				
Yes	183 (51.0%)	351 (32.6%)	2.151 (1.687–2.742)	<0.001
No	176 (49.0%)	726 (60.4%)	1.0	

OR = Odds Ratio, CI = Confidence Interval.

**Table 2:** Individual risk factors for mycetoma at Eastern Sennar locality, Sennar State (n = 1436).

Individual Characteristics	Cases No. (%)	Controls (%)	AOR (95% CI)	*p*-Value
**Age group**				
0–15 years	70 (19.5%)	70 (6.5%)	1.0	
16–30 years	136 (37.9%)	391 (36.3%)	2.804 (1.424–5.523)	0.003
31–45 years	101 (28.1%)	344 (31.9%)	1.564 (0.852–2.871)	0.149
46–60 years	37 (10.3%)	175 (16.2%)	1.469 (0.791–2.726)	0.223
>60 years	15 (4.2%)	97 (9.0%)	1.218 (0.617–2.405)	0.570
**Trauma history**				
Yes	129 (35.9%)	266 (24.7%)	1.892 (1.425–2.513)	<0.001
No	230 (64.1%)	811 (75.3%)	1.0	
**Marital status**				
Married	207 (57.7%)	914 (84.9%)	1.0	
Unmarried	152 (42.3%)	163 (15.1%)	3.179 (2.339–4.20)	<0.001
**Education level**				
Literate	220 (61.3%)	559 (51.9%)	1.0	
Illiterate	139 (38.7%)	518 (48.1)	0.685 (0.521–0.900)	0.007
**Animal raised within the dwelling**				
Yes	127 (35.4%)	534 (49.6%)	0.303 (0.220–0.416)	<0.001
No	232 (64.6%)	543 (50.4%)	1.0	
**Livestock ownership**				
Yes	183 (51.0%)	351 (32.6%)	3.941 (2.874–5.405)	<0.001
No	176 (49.0%)	726 (60.4%)	1.0	

AOR = Adjusted Odds Ratio, CI = Confidence Interval.

## Data Availability

The datasets used and/or analyzed during the current study are available from the corresponding author on reasonable request on the following email: roaalbasha2016@outlook.com.
